# Supplementary material for: WhereWulff: A Semiautonomous Workflow for Systematic Catalyst Surface Reactivity under Reaction Conditions
Source: J Chem Inf Model. 2023 Apr 5;63(8):2427–37. doi: 10.1021/acs.jcim.3c00142 (PMC10131224; doi:10.1021/acs.jcim.3c00142)
Supplement: Supplementary file 1 — ci3c00142_si_001.pdf [file ci3c00142_si_001.pdf]

Supporting Information: WhereWulff: A semi-autonomous workflow for systematic catalyst surface reactivity under reaction conditions

Rohan Yuri Sanspeur,[†,¶] Javier Heras-Domingo,[†,¶] John R. Kitchin,[†] and Zachary Ulissi[*,†,‡]

†*Department of Chemical Engineering, Carnegie Mellon University, 5000 Forbes Ave., Pittsburgh, Pennsylvania, 15213, USA*

‡*Scott Institute for Energy Innovation, Carnegie Mellon University, 5000 Forbes Ave., Pittsburgh, Pennsylvania, 15213, USA*

¶*These authors contributed equally to this work*

E-mail: zulissi@andrew.cmu.edu

Contents

S1 Non-stoichiometric Surface Energy Calculations **S-3**

S2 Surface Coverage and OER **S-4**

S3 Convergence Tests and Settings **S-6**

S4 De-prioritized Surface Energies **S-8**

S5 Surface Pourbaix Diagrams **S-9**

S6 DFT Figures $BaSnTi_2O_6$ **S-10**

S7 DFT Figures $BaSrCo_2O_6$ **S-14**

References **S-17**

S1 Non-stoichiometric Surface Energy Calculations

One can compute the surface energy of a symmetric slab:

$$\gamma = \frac{\left[G_{\text{slab}} - \sum_i N_i \mu_i\right]}{2A} \tag{1}$$

distributing it between the two surfaces, each having area A, where the sum is over the chemical potentials μ_i and the number of atoms N_i of each of the species that make up the composition of the slab, as shown in Eq. (1).

Assuming that the bulk is in equilibrium with the slab and by defining a reference, we can conveniently re-write Eq. (1) to be as a function of the bulk energy per formula unit, as shown in Eq. (2), where the last sum represents the free energy excess, x_i the number of atoms per bulk formula and N_{ref} is the reference specie that is picked.

$$\gamma = \frac{\left[G_{\text{slab}} - N_{\text{ref}} \cdot g_{\text{bulk}} - \sum_i \left(N_i - x_i \cdot N_{\text{ref}}\right) \cdot \mu_i\right]}{2A} \tag{2}$$

More concretely, the derivation of Eq. (2) for a slab consisting of N_{Ba}, N_{Ti}, N_O and a bulk with formula $BaTiO_4$:

- From first principles we can express the Gibbs energy of the system as the sum of the interfacial energy and the cost of introducing/removing elemental species$_i$ at some temperature (T) and pressure (p)

$$\begin{aligned} G(x_{Ba}, x_{Ti}, x_O, T, p) = \mu_{Ba}(T,p)N_{Ba} + \\ \mu_{Ti}(T,p)N_{Ti} + \\ \mu_O(T,p)N_O + \\ \gamma \cdot 2A \end{aligned} \tag{3}$$

- Rearranging Eq. (3) for the surface energy, γ, and assuming symmetric surfaces, we get Eq. (4)

$$\gamma = \frac{\begin{aligned}\left[G(x_{Ba}, x_{Ti}, x_O, T, p) - \mu_{Ba}(T,p)N_{Ba} - \right. \\ \left. \mu_{Ti}(T,p)N_{Ti} - \mu_O(T,p)N_O\right]\end{aligned}}{2A} \tag{4}$$

- If we assume that the bulk is in equilibrium with the slab, we can write Eq. (5)

$$g_{\mathrm{BaTiO_4}} = \left[x_{Ba}^{bulk}\mu_{Ba}(T,p) + x_{Ti}^{bulk}\mu_{Ti}(T,p) + x_{O}^{bulk}\mu_{O}(T,p)\right] \cdot N_{bulk} \tag{5}$$

- We define the reference to be in relation to Ba per Eq. (6)

$$\mu_{Ba} = \frac{1}{x_{Ba}^{bulk}}\left(\frac{g_{\mathrm{BaTiO_4}}}{N_{bulk}} - x_{O}^{bulk}\mu_{O}(T,p) - x_{Ti}^{bulk}\mu_{Ti}(T,p)\right) \tag{6}$$

- We then introduce the bulk energy per formula unit into Eq. (4) by substituting Eq. (6), to get Eq. (7)

$$\begin{gathered}\gamma = \tfrac{1}{2A}\left[G(x_{Ba}, x_{Ti}, x_O, T, p) - \tfrac{N_{Ba}}{x_{Ba}^{bulk}N_{bulk}}g_{\mathrm{BaTiO_4}} + \right. \\ \left. \mu_O(T,p)\left(\tfrac{x_O^{bulk}N_{Ba}}{x_{Ba}^{bulk}} - N_O\right) + \mu_{Ti}(T,p)\left(\tfrac{x_{Ti}^{bulk}N_{Ba}}{x_{Ba}^{bulk}} - N_{Ti}\right)\right]\end{gathered} \tag{7}$$

- Eq. (7) reduces to the well-known surface energy Eq. (8) when the stoichiometry between the bulk and the interface is maintained

$$\gamma = \frac{E_{slab} - n \cdot E_{bulk}}{2A} \tag{8}$$

The slab model is made up of a 2D surface and a corresponding oriented bulk, since this has been shown to most efficiently converge the surface energy calculations.[S1]

S2 Surface Coverage and OER

The coverage effects of reaction intermediates (OH*, O*) may significantly impact the local environment of the active site, resulting in changes in the adsorption strength of the OER reaction intermediates. Consequently, it is important to determine the most stable surface coverage at given conditions of applied potential and pH to more reasonably describe the OER activity. To determine the most stable surface coverage, Surface Pourbaix diagrams,[S2] were constructed at three extreme coverages, clean, OH*, and O* terminated.

OER catalytic activities of the different surface structures were determined by the theoretical

overpotential (η_{OER}) and the potential-determining step (PDS) by assuming an associative reaction mechanism with O*, OH* and OOH* as reaction intermediates. The four proton-coupled electron transfers (PCET) reactions under acidic conditions are:

$$H_2O + (*) \longrightarrow OH^* + (H^+ + e^-) \tag{9}$$

$$OH^* \longrightarrow O^* + (H^+ + e^-) \tag{10}$$

$$O^* + H_2O \longrightarrow OOH^*(H^+ + e^-) \tag{11}$$

$$OOH^* \longrightarrow O_2\uparrow + (*) + (H^+ + e^-) \tag{12}$$

The computational hydrogen electrode (CHE) was used to express the chemical potential of the proton-electron pair ($H^+ + e^-$), which is related to the chemical potential of H_2 based on the equilibrium $\mu[H^+] + \mu[e^-] = 0.5\ \mu[H_2(g)]$ at 0 V_{RHE} (Reversible Hydrogen Electrode) and corrects the driving force with the deviation of the applied potential from the equilibrium situation. To avoid the use of O_2 electronic energy, which is difficult to determine correctly within standard GGA-DFT, the experimental free energy of $2\,H_2O \longrightarrow O_2 + 2\,H_2$ ($\Delta G = 4.92$ eV), was used. Therefore, the Gibbs free energies of reactions (9)-(12) depend on the adsorption free energies of the reaction intermediates (ΔG_{OH*}, ΔG_{O*}, and ΔG_{OOH*}) which are calculated relative to H_2O(g) and H_2(g) at $U = 0$ V and standard conditions. In total, 133 adsorption calculations were performed to calculate the adsorption free energies of the reaction intermediates, considering multiple orientations for OH* and OOH* on the surface termination for all the materials, and selecting the most stable one in each case.

The theoretical thermodynamic OER overpotential, which is a measure of the activity of a catalyst, is then defined from Gibbs free energies of reactions (9)-(12):

$$\eta_{OER}(V) = max[\Delta G_{\mathrm{OH}}, \Delta G_{\mathrm{O}} - \Delta G_{\mathrm{OH}}, \Delta G_{\mathrm{OOH}} - \Delta G_{\mathrm{O}}, 4.92 - \Delta G_{\mathrm{OOH}}]/e - 1.23 \tag{13}$$

The step with the largest value in Eq. (13) is referred to as the potential-determining step (PDS). It is important to note that the overpotential should not be compared directly with a measured

overpotential, since the measured overpotential depends on the current density.

S3 Convergence Tests and Settings

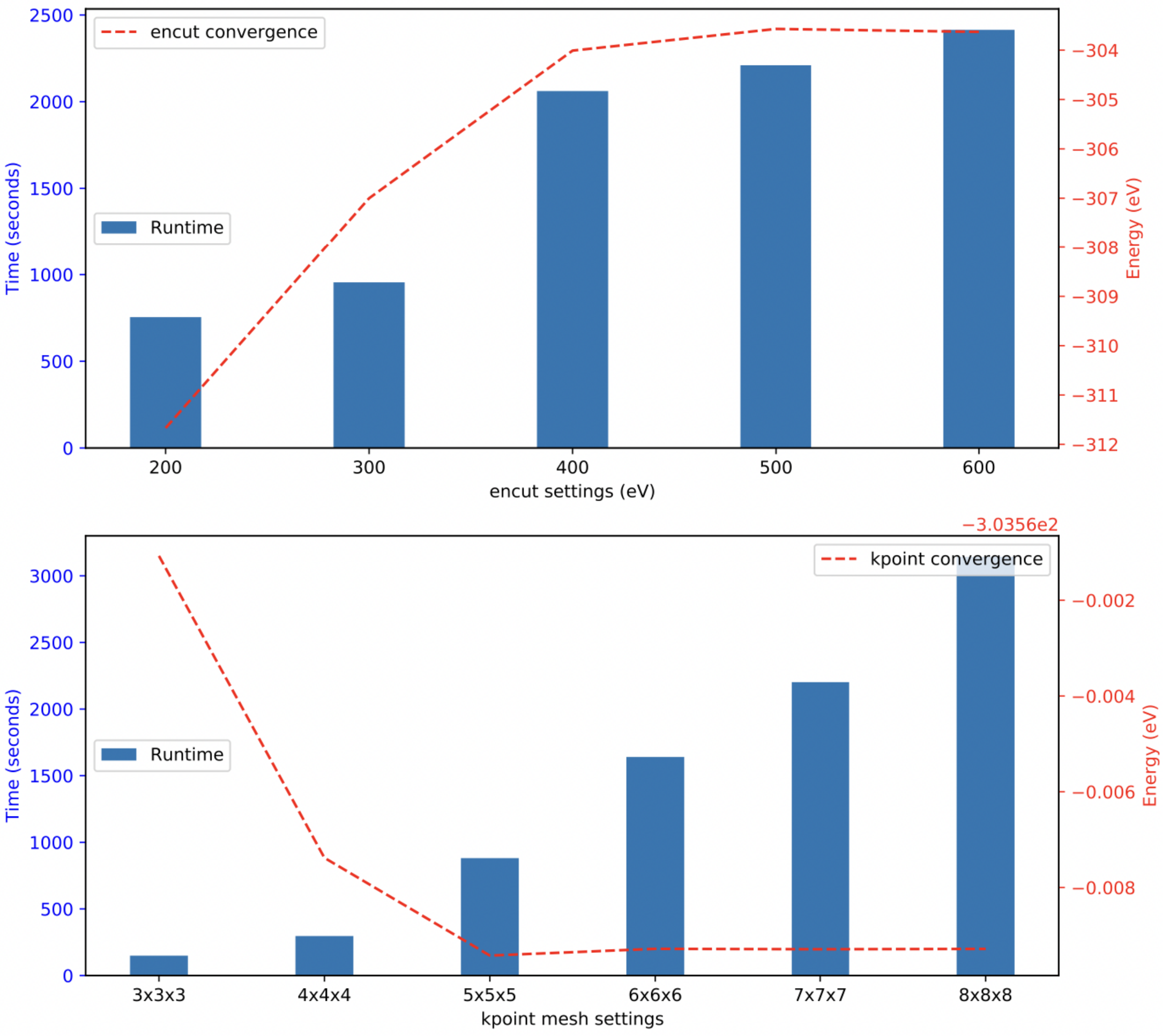

Figure S1: Standard convergence tests on bulk $BaSnTi_2O_6$ illustrating the trade-off between accuracy and computational time. Based on these results, we infer that using an ENCUT setting of 500 eV and a kpoint grid of 5×5×5 would be a good balance between accuracy and runtime. Our custom DFT settings, implemented as a child class of Pymatgen's *MVLSlabSet*, obey these thresholds.

Table S1: Slab thickness convergence table showing how we settled on a slab thickness of 3 in units of oriented unit cell under a (2×1) supercell. Since the surface energy changes within 1.24 $\frac{meV}{\AA^2}$ from a slab thickness of 3 to 4 and the runtime cubically in the number of electrons, we compromise on a slab thickness of 3 consistently throughout our case study.

Slab Thickness (multiple of oriented unit cell)	Slab Energy (eV)	$\gamma_{(hkl)}$ $(\frac{J}{m^2})$	Runtime (days)	Number of atoms
1	-308.70	NA	0.12	40
2	-610.81	0.60	3.79	80
3	-919.93	0.56	15.73	120
4	-1228.37	0.58	16.89	160
5[a]	-1536.79	0.61	15.79	200

[a] This slab optimization did not converge in the allotted number of re-submissions (10). These results corroborate that it is infeasible for us to carry out the slab optimizations at those levels of thickness and under such large numbers of degrees of freedom, which are shown to trigger multiple re-submissions (Figure S2).

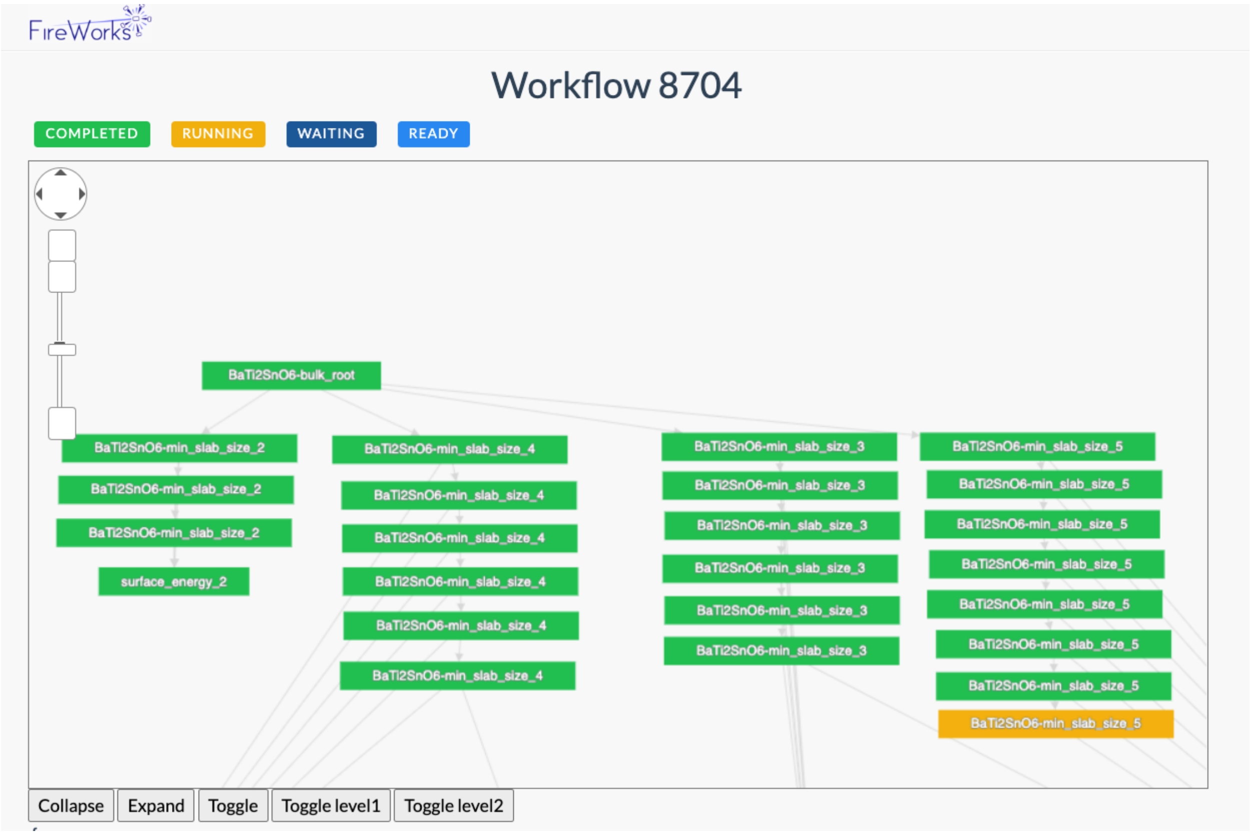

Figure S2: Fireworks[S3] web-gui showing how we repurposed some of the components of *WhereWulff* to conduct slab thickness convergence tests.

S4 De-prioritized Surface Energies

Table S2: De-prioritized surface energies in J/m^2.

Formula	(hkl)	$\gamma_{(hkl)}$
$Ba_5Sr_5(Co_6O_{17})_2$	(100)	0.74
$Ba_5Sr_5(CoO_3)_{12}$	(100)	0.94
$Ba_3Sr_3Co_6O_{17}$	(101)	0.65
$Ba_5Ti_{12}Sn_5O_{34}$	(100)	0.45
$Ba_5Ti_{12}Sn_5O_{36}$	(100)	0.34
$Ba_3Ti_6Sn_3O_{17}$	(101)	2.20

S5 Surface Pourbaix Diagrams

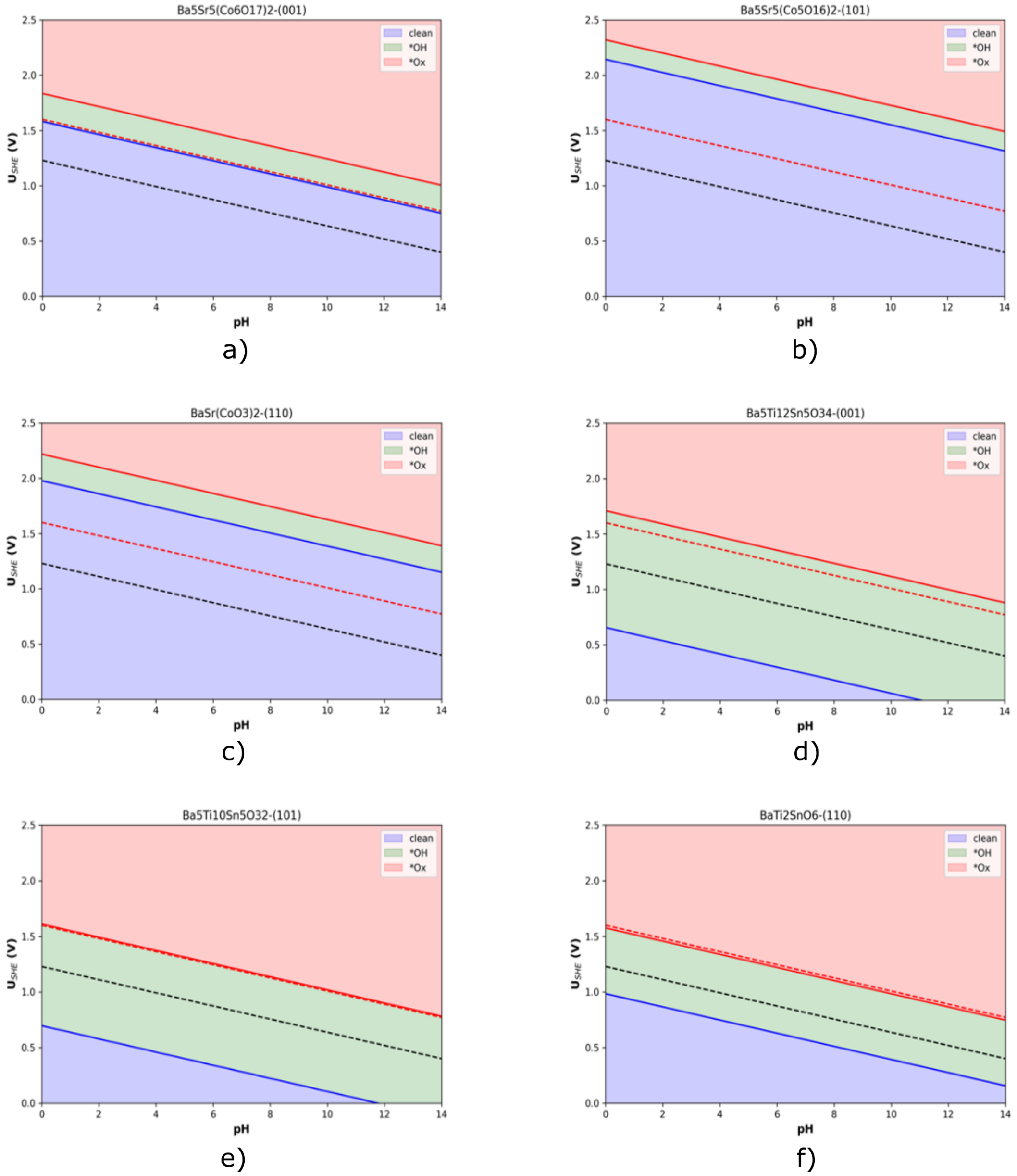

Figure S3: Surface Pourbaix Diagrams across all materials and facets studied in this work.

S6 DFT Figures $BaSnTi_2O_6$

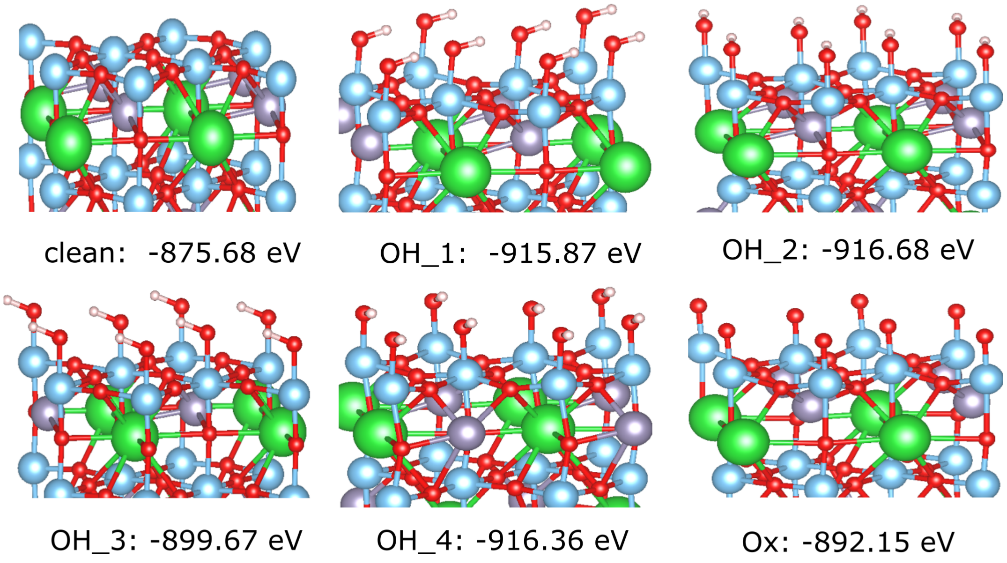

Figure S4: $Ba_5Ti_{12}Sn_5O_{34}$−(001) surface Pourbaix Diagram Intermediates: From Clean (Top-Left), OH* rotation screening (1-4) and O*.

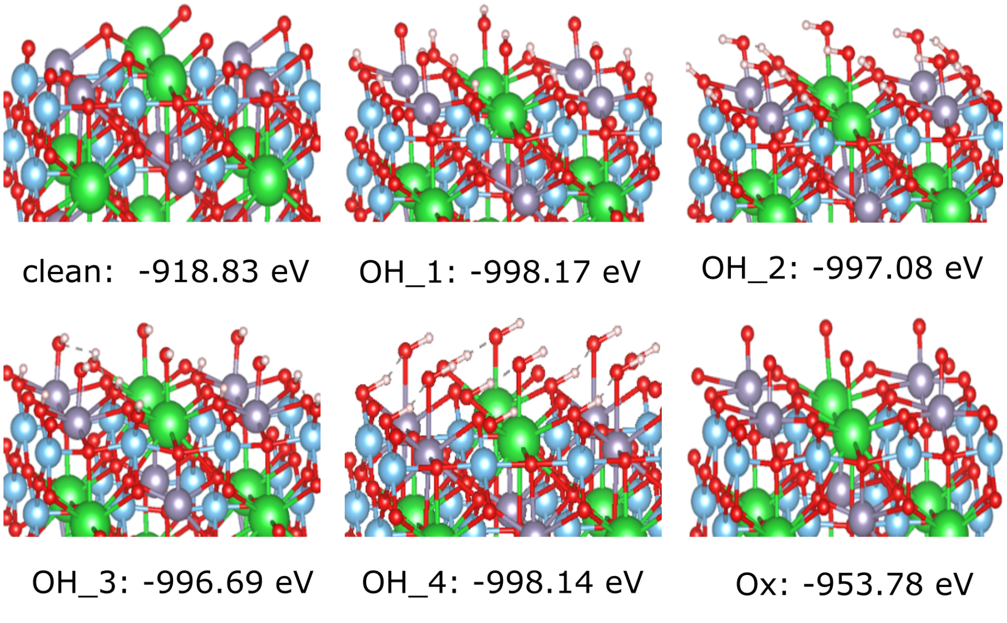

Figure S5: $BaTi_2SnO_6$−(110) surface Pourbaix Diagram Intermediates: From Clean (Top-Left), OH* rotation screening (1-4) and O*.

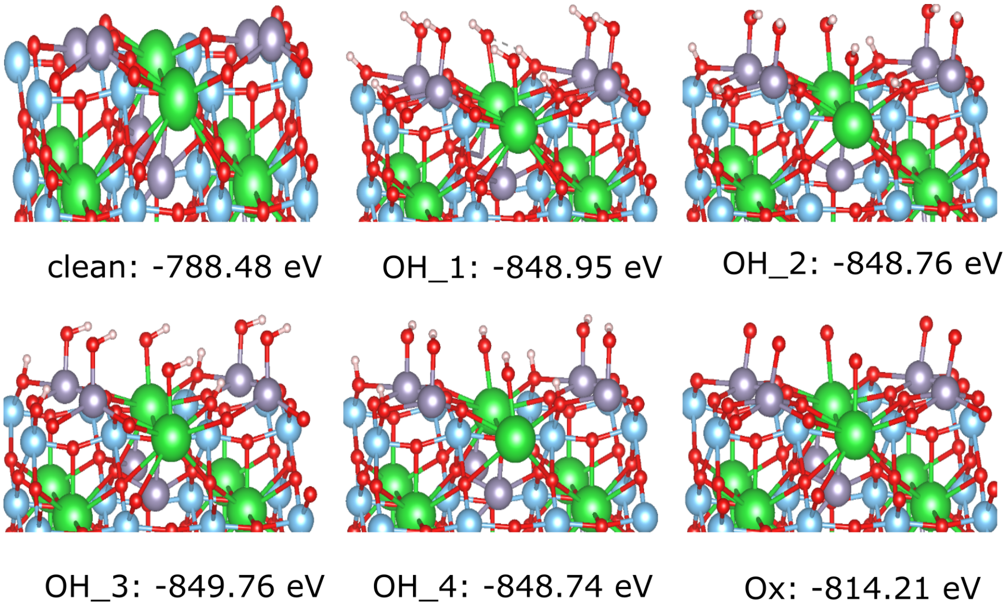

Figure S6: $Ba_5Ti_{10}Sn_5O_{32}$−(101) surface Pourbaix Diagram Intermediates: From Clean (Top-Left), OH* rotation screening (1-4) and O*.

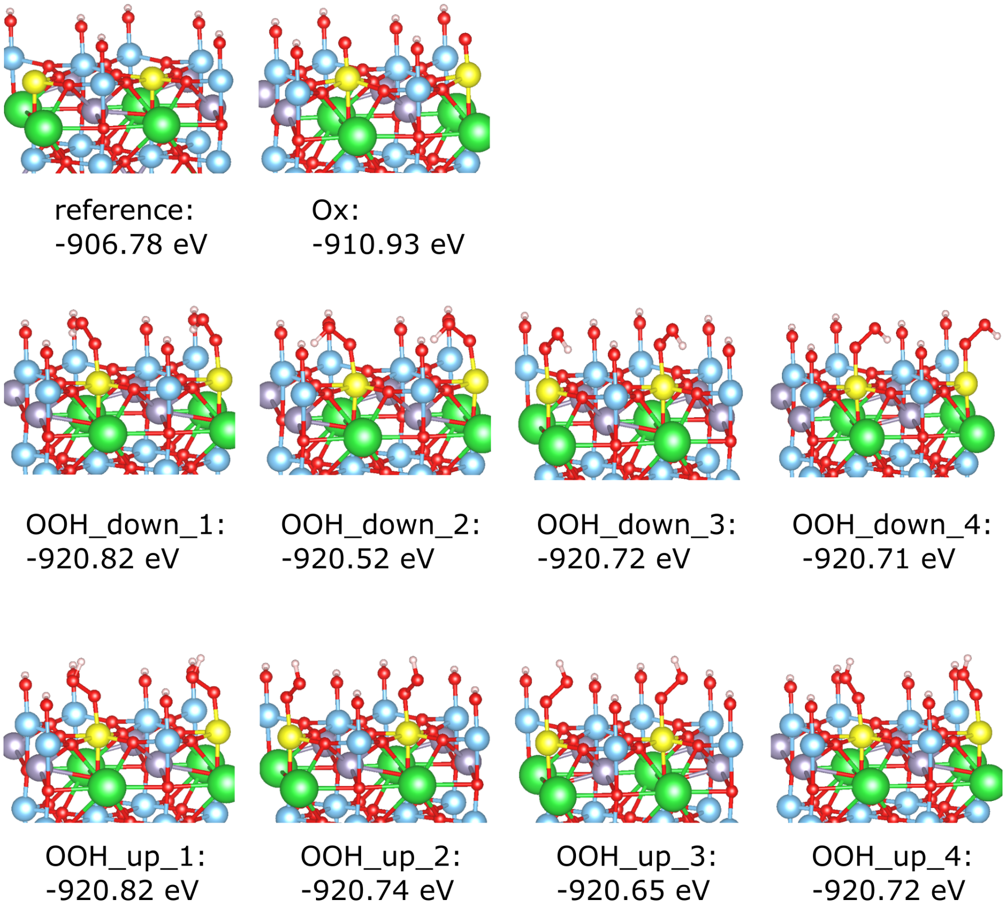

Figure S7: $BaSnTi_2O_6$ Reactivity for the *OH terminated (001).

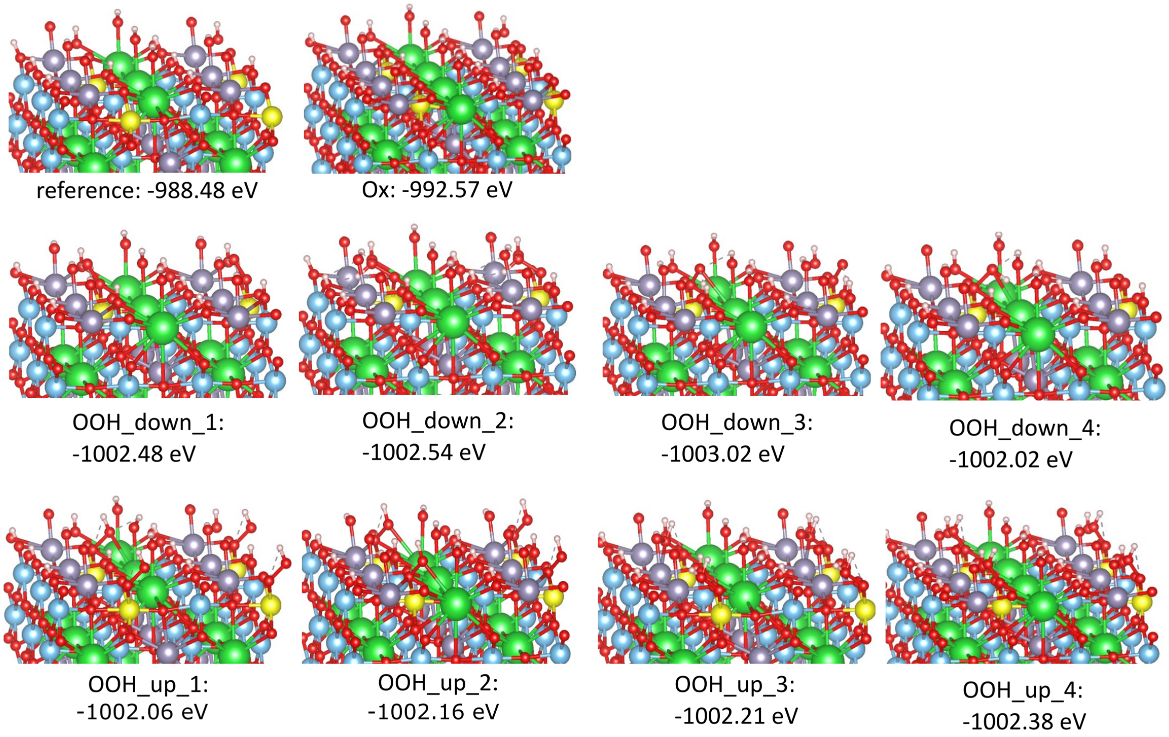

Figure S8: $BaSnTi_2O_6$ Reactivity for the *OH terminated (110) surface.

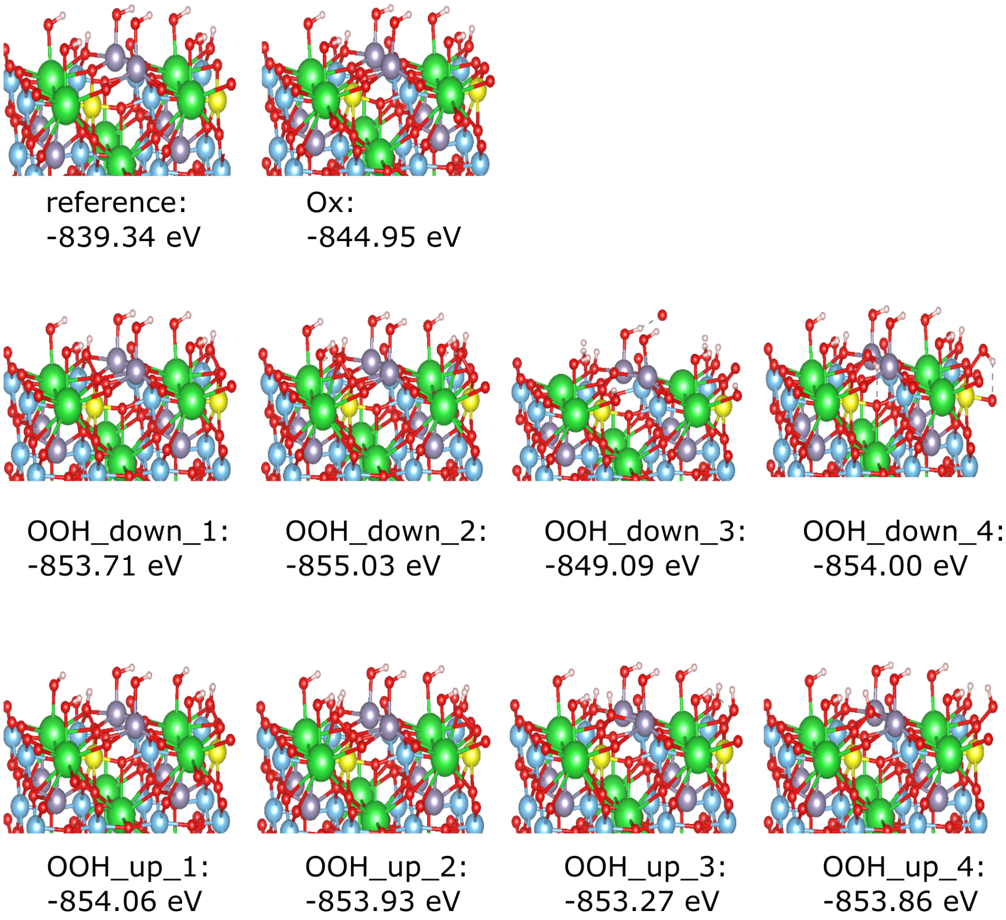

Figure S9: $BaSnTi_2O_6$ Reactivity for the *OH terminated (101) surface.

S7 DFT Figures $BaSrCo_2O_6$

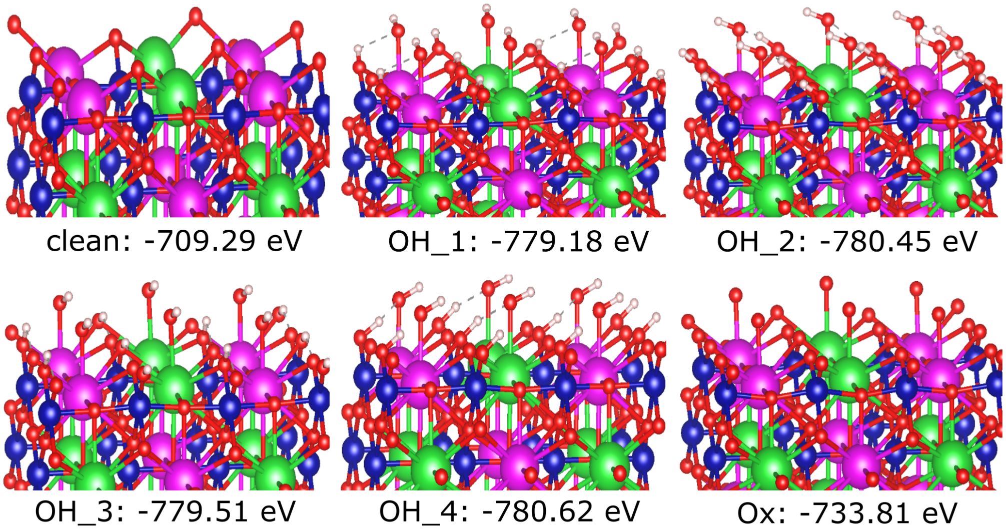

Figure S10: $BaSr(CoO_3)_2-(110)$ surface Pourbaix Diagram Intermediates: From Clean (Top-Left), OH* rotation screening (1-4) and O*.

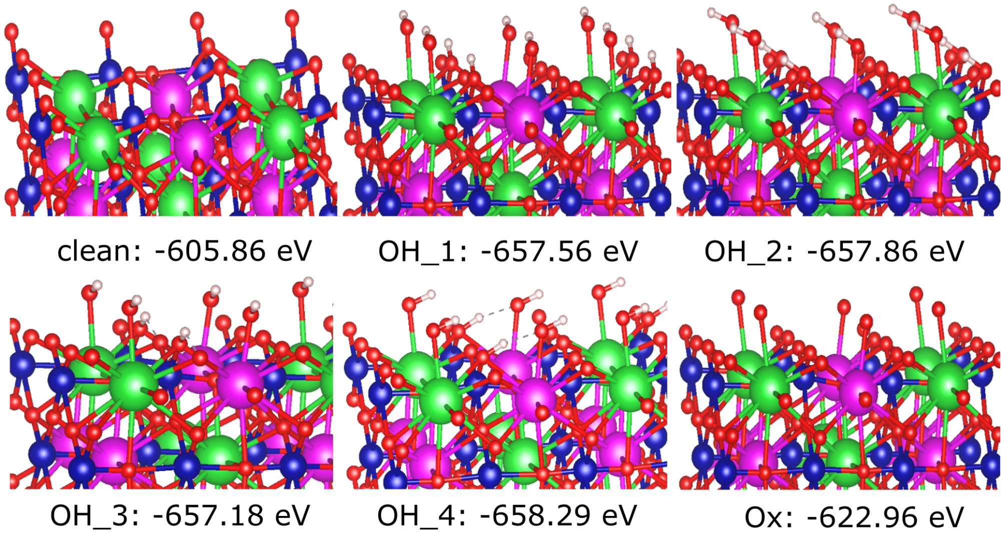

Figure S11: $Ba_5Sr_5(Co_5O_{16})_2-(101)$ surface Pourbaix Diagram Intermediates: From Clean (Top-Left), OH* rotation screening (1-4) and O*.

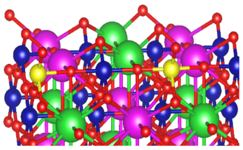

reference:
-709.29 eV

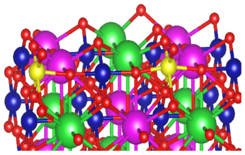

Ox:
-713.33 eV

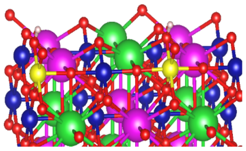

OH_1:
-719.21 eV

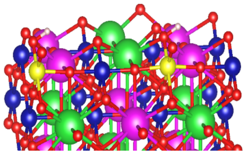

OH_2:
-719.04 eV

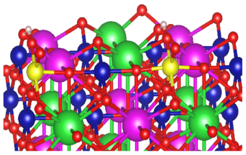

OH_3:
-718.39 eV

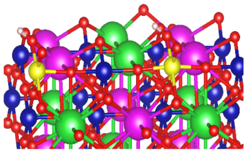

OH_4:
-718.98 eV

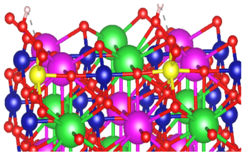

OOH_up_1:
-722.91 eV

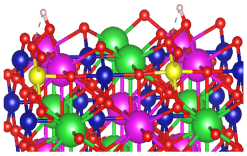

OOH_up_2:
-723.10 eV

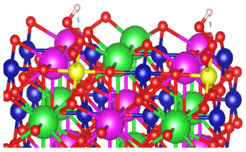

OOH_up_3:
-723.25 eV

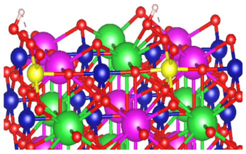

OOH_up_4:
-723.20 eV

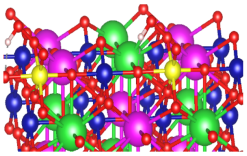

OOH_down_1:
-723.29 eV

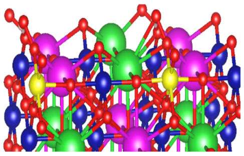

OOH_down_2:
-723.62 eV

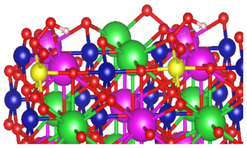

OOH_down_3:
-722.91 eV

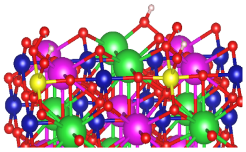

OOH_down_4:
-724.41 eV

Figure S12: $BaSr(CoO_3)_2$ Reactivity for the clean (110) termination.

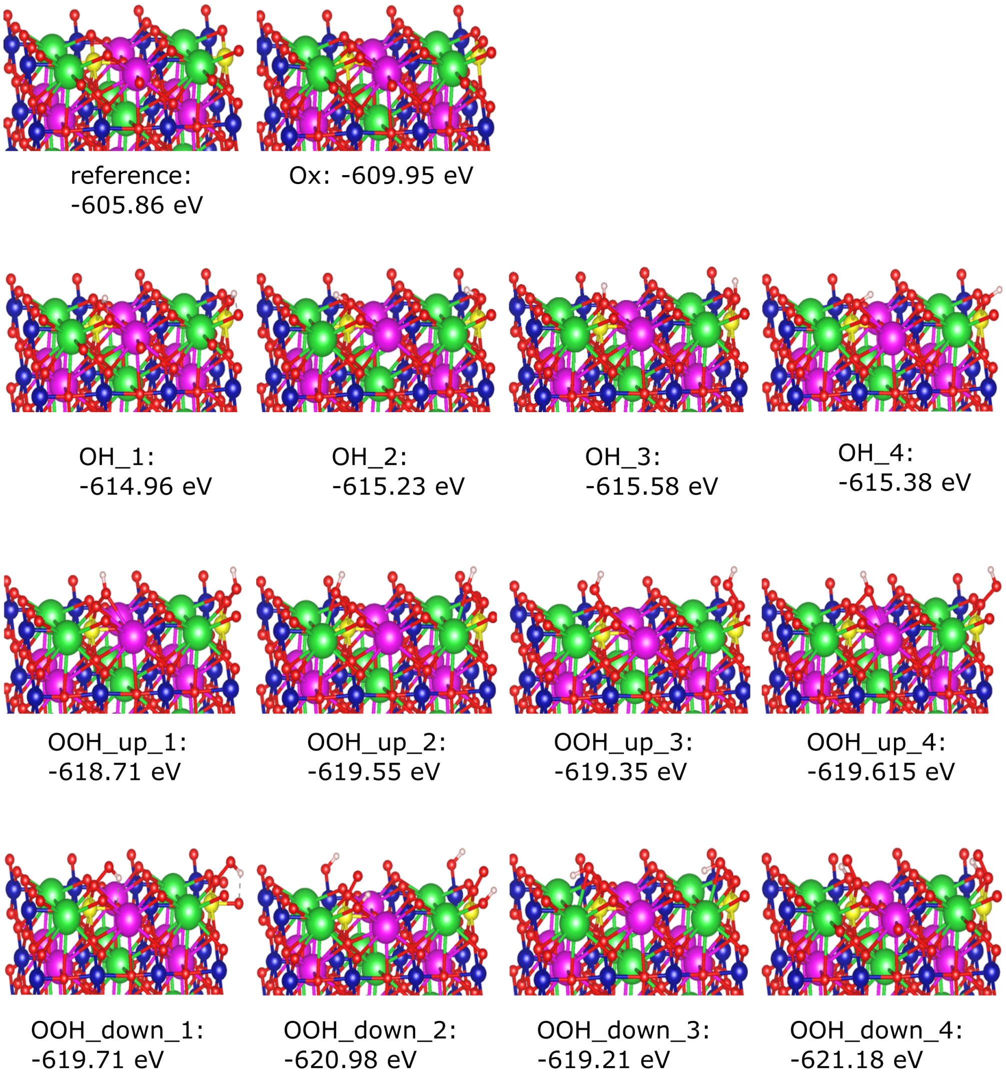

Figure S13: $BaSr(CoO_3)_2$ Reactivity for the clean terminated (101).

References

(S1) Sun, W.; Ceder, G. Efficient creation and convergence of surface slabs. *Surf. Sci.* **2013**, *617*, 53–59.

(S2) Sumaria, V.; Krishnamurthy, D.; Viswanathan, V. Quantifying Confidence in DFT Predicted Surface Pourbaix Diagrams and Associated Reaction Pathways for Chlorine Evolution. *ACS Catal.* **2018**, *8*, 9034–9042.

(S3) Jain, A.; Ong, S. P.; Chen, W.; Medasani, B.; Qu, X.; Kocher, M.; Brafman, M.; Petretto, G.; Rignanese, G.; Hautier, G.; Gunter, D.; Persson, K. FireWorks: a dynamic workflow system designed for high-throughput applications. *Concurrency and Computation: Practice and Experience* **2015**, *27*, 5037–5059.
